# Onchocerciasis, an undiagnosed disease in Mozambique: identifying research opportunities

**DOI:** 10.1186/s13071-016-1468-7

**Published:** 2016-03-31

**Authors:** Emilia V. Noormahomed, Kevan Akrami, Carmen Mascaró-Lazcano

**Affiliations:** Department of Microbiology, Universidade Eduardo Mondlane, Maputo, Mozambique; Department of Medicine, University of California, San Diego, CA USA; Universidade Lúrio, Reitoria, Bairro de Marrere, Rua 4250, Km 2,3, Caixa Postal 360 Nampula, Moçambique; Department of Parasitology, Universidad de Granada, Granada, Spain

**Keywords:** Mozambique, Onchocerciasis, Onchocerca volvulus, Scabies, Leprosy

## Abstract

**Background:**

The objective of this paper is to summarise and critically review the available data about onchocerciasis in Mozambique, in order to report epidemiological and clinical aspects related to the disease and identify gaps in knowledge. The paper is intended to raise awareness of the existence and importance of this disease and to define research priorities.

**Methods:**

We examined the scarce epidemiological data at our disposal: two diagnostic studies in 1997 and 1998 (first reports on the existence of onchocerciasis in Mozambique), and two Rapid Epidemiological Mapping of Onchocerciasis (REMO) surveys in 2001 and 2007. We examined differences in study designs and methodologies as well as the differing geographical locations to explain the divergence in findings among the studies.

**Results:**

Evidence indicates that onchocerciasis is hypoendemic in Mozambique (with national and imported cases), but still largely remains an undiagnosed illness. There is no awareness of the clinical aspects of the disease and nor of the differential diagnosis with lepromatous leprosy and dermatitis caused by *Scabies* spp. The use of skin biopsy and a symptom screening questionnaire, combined with nodule rate, in the first two studies may have captured even atypical or subacute presentations. Both REMO surveys relied solely on nodule detection and in the six years between the two studies, the prevalence of nodules detected more than doubled.

**Conclusions:**

The epidemiology and clinical aspects of the disease are unknown in Mozambique. Since the last REMO took place in 2007 and since the population is subject to large-scale movement and displacement, it is important to develop tools to identify and analyse populations that are at high risk for onchocerciasis. Cases of onchocerciasis may be misdiagnosed as leprosy or scabies that fail to improve despite being subjected to treatment against leprosy. Techniques to enable a differential diagnosis need to be established by training health professionals on the recognition of this undiagnosed disease. It is equally necessary to identify the blackfly vectors and where they breed.

## Background

Onchocerciasis caused by *Onchocerca volvulus* is a chronic cutaneous and ocular disease characterised by skin nodules, severe itching, and ocular lesions that can progress to partial or total blindness. It causes a great morbidity and is the second leading cause of blindness due to infectious disease worldwide. More than 99 % of affected people live in Africa. It has been estimated that 21,115,000 members of the 118,285,000 African population at risk for infection are infected. Among these, 690,000 experienced visual impairment and 220,000 were totally blind [1].

The vectors are dipterans of the family Simulidae, known as blackflies, buffalo gnats or turkey gnats, although there are more than a hundred other local names throughout Africa. At least 26 members of the *Simulium damnosum* Theobald complex are known in southern and eastern Africa [2]. Blackflies become infected by feeding on a person with microfilariae (mf) in the skin (microfiladermia). Microfilariae develop to third stage larvae, migrating to the blackfly labium and can penetrate the human skin through the fly bite. They become adult in about one year, usually within the subcutaneous nodules (onchocercomas) (0.5–10 cm or larger). The onchocercomas include bundles of worms located in the subcutaneous connective tissue, the juxta-articular areas, and rarely in the muscles or bones. They are usually painless, hard, and fibrous, mobile under the fingers when palpated and rarely calcify. A patient may have only one or many onchocercomas. Adult parasites can be also found free under the subcutaneous tissue. Several authors indicate that in African patients nodules are located mainly on the trunk (especially on the hip area) around the pelvis, particularly over the iliac crest, femoral trochanter, coccyx, sacrum and lateral chest wall, and on the limbs. But nodules can also be found in the upper body, even in the head with percentages that can reach over 30 %. Many head and upper body nodules probably remain undetected in onchocerciasis surveys [3].

Onchocerciasis is a systemic disease, as mf are distributed throughout the body; they can be found not only in skin, eyes and lymph nodes, but also in the liver, kidney, spleen, pancreas, lung, peripheral nerves and arteries. Body fluids like blood, urine, hydrocele and vaginal fluids, tears, cerebrospinal fluid and peritoneal may contain microfilariae [4]. An association between microfiladermia and backache, body and joint pains has been described; it also seems clear that persons with microfiladermia weigh less than those without [5]. Highly infected onchocerciasis patients are more likely to develop the lepromatous form of leprosy, suggesting a striking impairment of cell-mediated immunity [6].

An association of onchocerciasis and epilepsy was first suggested in 1994 in Uganda [7], later in other African countries. A meta-analysis including a total population of 79,270 individuals from ninety-one communities living in seven countries (Benin, Nigeria, Cameroon, Central African Republic, Uganda, Tanzania and Burundi) concluded that onchocerciasis was associated with epilepsy [8]. Onchocerciasis has also been associated with nodding syndrome (NS), an epidemic epileptic encephalopathy affecting thousands of children in Uganda, South Sudan and Tanzania [9].

The psychological effects of onchocerciasis are also very important as dermal lesions and lymph disorders stigmatise and marginalise patients. The three components of human health, physical, mental and social, are strongly affected by this disease. Their individual and social effects have been studied in communities of south Nigeria [10]. A study revealed that onchodermatitis was the main health problem of teenage Nigerian girls as it reduced or eliminated their chances to marry [11].

The clinical manifestations of onchocerciasis are divided into dermal changes, lymphatic alterations and ocular lesions. The prepatent period ranges from 3 to 15 months. While the microfilariae still are alive under the subcutaneous tissue they do not trigger an inflammatory reaction. Itching is the first manifestation (isolated or associated with skin lesions) that may be troublesome and even irresistible, and scratching leads to a picture of filarial scabies as it can be confused with scabies, especially in countries where suspicion of onchocerciasis does not arise. In Africa these lesions mainly affect the lumbar region, buttocks, thighs and legs. In African patients there are two major changes. In the so-called “lizard skin”, the skin dries, hardens and peels (keratin layers), and the elastic fibres of the dermis are lost thus giving the appearance of ageing. “Leopard skin” occurs in chronic patients, where loss of melanin leads to large depigmented spots with black dots indicating hair follicles and pores, particularly patent on the calfs. Lymphatic manifestations include swollen lymph glands, lymphadenitis, hanging groin due to chronic inflammation and fibrosis that impair the lymph circulation. Complications include hernias (inguinal or femoral), and usually affect the scrotum. Some atypical clinical manifestations have been described such as abscesses around the anus or in the groin [12, 13].

Ocular manifestations range from mild to total blindness (white eyes). They can affect any area of the eye and are only reversible in the initial phase of the ocular invasion. Early signs are night blindness (hemeralopia), and photophobia, with a bilateral decrease in daytime vision.

The discovery of *Wolbachia* and its importance in the development of symptoms in onchocerciasis [14] raised the possibility of eliminating the bacteria with antibiotics to indirectly kill the filariae. The use of tetracyclines (doxycycline) was demonstrated as effective [15], but requires four to six weeks of treatment and is contraindicated in children, pregnant or breastfeeding women. With the aim to develop new drugs against onchocerciasis and other filariasis the “Anti-*Wolbachia*” consortium was created in 2007 [16].

The clinical diagnosis of onchocerciasis is based on physical examination for onchocercomas, and the presence of skin, lymph and eye disorders. The onchocercomas are only detectable by palpation over flat bones; to detect those at deeper locations imaging techniques are required. The low sensitivity of nodule palpation indicated that is an unreliable method in areas with low prevalence; the value of this method is too low to be capable of supporting an epidemiological decision [17]. In experimentally infected *Pan troglodytes* many adult *O. volvulus* lie in deep tissues where they are impalpable. Sites of preference were posterior to the hip joint, adjacent to the joint capsule, against the shaft of the humerus and near the lesser trochanter [18].

In African patients skin biopsy from the buttocks, not popular among patients, is recommended for diagnosis of microfiladermia. It has limitations since in advanced and chronic cases the microfilariae are in deep layers of the dermis, therefore leading to false negatives. The Mazzoti test is an indirect diagnosis that involves giving the patient a small dose of ivermectin and noting adverse reactions. It was replaced by the less unpleasant OCP-patch with DEC (diethylcarbamazine) that induces a local inflammatory reaction. A new developed ready-to-use format (LTS-2 patch) avoids the previous labour-intensive preparation allowing large-scale use [19] Ultrasonography (USG) has been evaluated for the detection of changes in the onchocercomas after treatment [20], and may be used as an adjunct to histological examination as it can even pick up adults moving within them. Enzyme linked immunosorbent assay (ELISA) for antibody detection is unable to distinguish an infection that has already been eliminated from one that is active; however, it can be effectively used to monitor the success of onchocerciasis elimination programs in Africa [21].

Rapid-format antibody cards for IgG4 which require only a drop of blood showed a sensitivity of 90.6 % [22]. It is a fast procedure, easy to use, and less painful for patients than the skin-snip test. Various molecular diagnostic techniques have been developed, qPCR in dried skin snips proves to be more sensitive than skin biopsy, but the two tests should be combined to be certain [23].

Control is based almost exclusively on large-scale treatment with ivermectin (Community Directed Treatment with Ivermectin, CDTI) given annually or every six months to the eligible members of the high risk communities. Although it was once believed that this measure would be insufficient, elimination has been achieved by long term CDTI alone in villages of Senegal, Mali and Sudan, reviving debate on the eradicability of onchocerciasis in Africa [24]. There are also studies showing the ineffectiveness of monitoring strategies despite repeated ivermectin treatment and vector control [25]. Clinical trials have demonstrated that moxidectin can be a potential alternative to ivermectin for its higher and prolonged efficacy, which results in almost full, year-long suppression of microfilaridermia [26].

Mozambique has an estimated population of more than 27 million and is divided into 11 provinces. Primary health care is delivered through rural health centres that typically cover an area of eight square kilometres, though mobile healthcare agents may cover more than 50 square kilometres. Staffed by one or two general physicians, they cover 165 populations of 50,000 to 900,000 and provide basic laboratory services and simple radiology. Some centres have basic laboratory services, such as faecal examination for parasite infection detection. None of these clinics have staff trained to perform skin biopsies to confirm the diagnosis of onchocerciasis, since it is an under-recognised problem in the country. Secondary health care is delivered by district hospitals, to which more complex cases are referred. There is no surveillance or control programme for onchocerciasis. The disease is well reported in neighbouring Tanzania and Malawi with 3,437,030 and 2,215,041 people, respectively, requiring preventive chemotherapy in 2014 according to the WHO. Areas surrounding Rivers Milange and Muloza in Malawi serve as breeding sites for *Simulium damnosum* [27]. In the districts of Thyolo and Mwanza, onchocerciasis prevalences in the past were 29.4 % and 28.5 %, respectively [28, 29].

The objective of this paper is to summarise and critically review the available data about onchocerciasis in Mozambique, to raise awareness of the existence and importance of the disease, and to define research priorities in terms of epidemiology, diagnosis, clinical manifestations, treatment, control, and economic and social impact of this disease.

## Methods and Results

### The first surveys

The first report on the existence of onchocerciasis in Mozambique was done by Noormahomed & Pividal in 1997 [30] and sponsored by the TDR-WHO in order to confirm the presence of this parasitic condition in the country and the possible occurrence of autochthonous transmission in the Milange District (Zambezia Province). There were two main reasons for choosing Milange District as a pilot study site: (i) Milange District borders Malawi along the Muloza River for 223 km and is known to be the breeding site for the vectors *Simulium* spp.; and (ii) during the civil war that affected Mozambique from 1976 to 1992 there was a migration of populations from Zambezia Province into Malawi, where endemic regions of onchocerciasis, were identified [28, 29].

In Milange District 316 people were recruited at the rural hospital of Milange. Most (98 %) of those studied were males over 20 years of age. After performing a clinical examination of the entire body, two skin snips were taken from the posterior iliac crest, using a Holth sclerocorneal punch, and placed immediately in microtiter plates with 200 μl of isotonic saline (NaCl 0.9 %).

Microscopic readings were made after 6 and 12 h and the number of microfilaria per field were counted. A symptom screening questionnaire was also completed by all participants. Of the total sample, 45 individuals were found to be infected, equivalent to an overall prevalence of 14 %. Three of the 45 positive cases denied ever having been in Malawi. The overall intensity of infection was light; 7.5 expressed by the mean number of microfilariae per mg of skin. These preliminary screening results confirmed for the first time that both autochthonous and imported onchocerciasis exists in Mozambique, and, as such, represented a potentially significant public health problem.

A second study (previously unpublished) was undertaken in 1998 by Dr E. Noormahomed in different villages of Milange (Zambezia Province) and in the Mecanhelas District (Niassa Province), also bordering Malawi. The aim of this follow-up study was to better clarify the variance in clinical presentation of onchocerciasis and the differences related to the epidemiological and parasitological findings. After obtaining informed consent 370 people were screened in 6 different villages. Similar to the first study, the screened individuals were adults over 20 years of age, the majority being male. Of those 141 (38.1 %) were not refugees. The screening showed that 196 (53 %) patients were positive for *O. volvulus* microfilariae based on skin biopsy, 120 (32.4 %) had visible or palpable nodules and 102 (27.5 %) expressed complaints about visual impairment (Table 1).

**Table 1:** Results obtained in the villages of Mozambique screened for *O. volvulus* in 1998 by E. Noormahomed

Village	People examined	Skin nodules^a^	Positive skin biopsy^b^	Visual impairment^c^	Natives of Mozambique^d^
Chizongo	120	33 (27.7)	60 (50.0)	26 (21.7)	34 (28.3)
Mambucha	44	15 (34.1)	26 (59.1)	15 (34.0)	6 (13.6)
Nambuzi	36	7 (19.4)	19 (52.8)	10 (27.7)	33 (91.6)
Vulalo	57	29 (51.0)	32 (56.1)	11 (19.3)	12 (21.0)
Matage	42	16 (38.1)	22 (52.3)	16 (38.1)	2 (4.7)
Mecanhelas	71	17 (24.0)	35 (49.3)	16 (22.5)	49 (69.0)
Total	370	120 (32.4)	196 (53)	102 (27.5)	141 (38.1)

^a^Number (%) of people with nodules; ^b^Number (%) of people showing microfilaria in skin biopsy; ^c^Number (%) of people that expressed complains about visual impairment; ^d^Number (%) of people who are not refugees from other countries

**Fig. 1:**
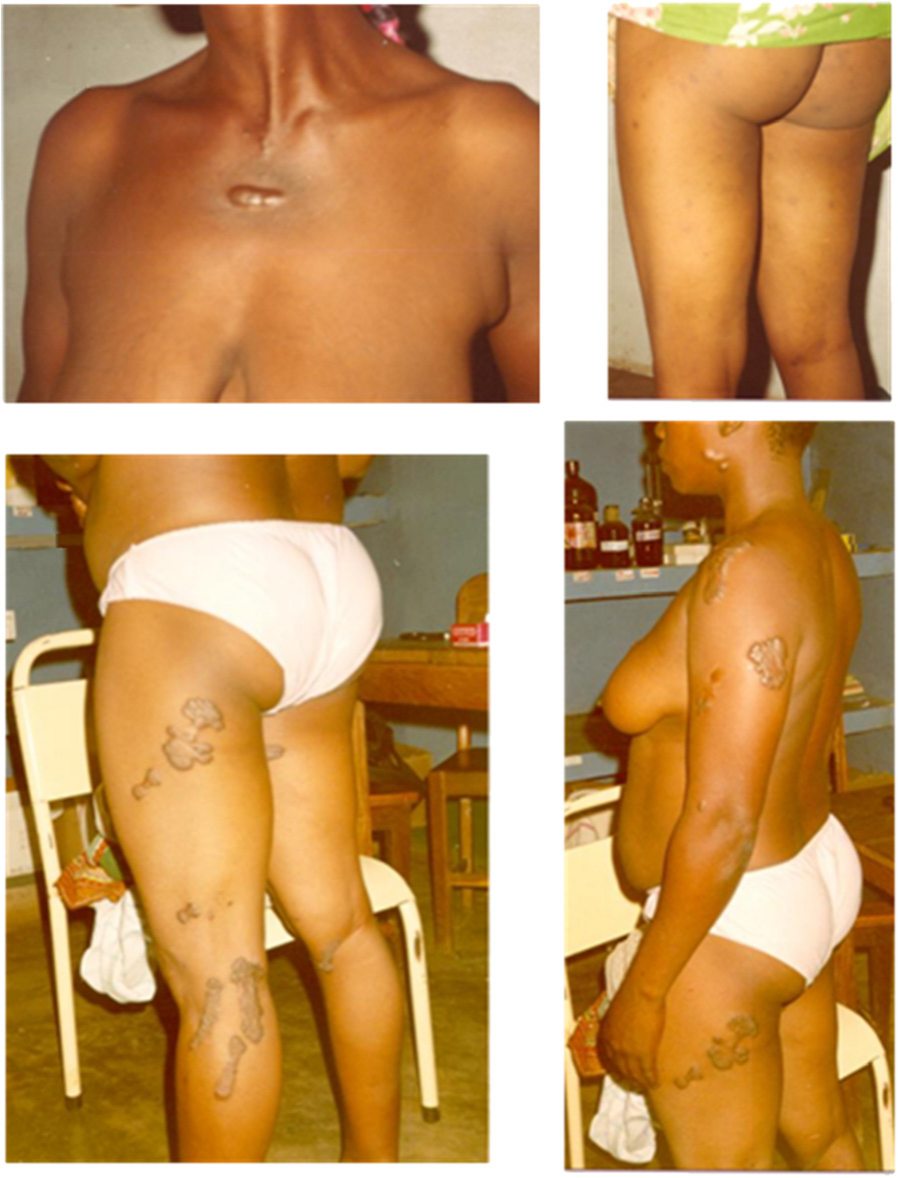
Some microfiladermia-positive individuals exhibited atypical dermal findings with erythematous plaques of irregular contour irregularly distributed on the trunk, extremities, and even on the face

The patients screened exhibited typical findings for onchocerciasis infection: the characteristic skin nodules were variably present above and below the hip, with skin depigmentation and dermatitis resembling scabies. Atypical dermal findings showing erythematous plaques of irregular contour distributed in the body are displayed in Fig. 1; investigation of these unusual manifestations was considered necessary. This screening clearly indicated that the detection of nodules by palpation is an inadequate method for diagnosis since 196 (53 %) patients were detected by skin biopsy, of which only 120 (32.43 %) showed palpable nodules. Thus, 38.7 % of cases (76 patients) would have remained undiagnosed if we had only used palpation as a method of detection.

### REMO surveys

Based on these preliminary data, the African Programme for Onchocerciasis Control (APOC) sponsored the first REMO in 2001 [31] in order to have a clear understanding of the distribution and endemicity level of the disease in Mozambique, so that appropriate measures could be implemented. A total of 282 villages were selected from eight out of the ten provinces in Mozambique, namely: Niassa, Cabo Delgado, Nampula, Zambezia, Tete, Manica, Sofala and Inhambane, according to the REMO protocol (and other technical criteria) since the population has been migrating in and out of neighbouring countries over the last 2–3 decades due to war.

REMO was successfully carried out in 198 of the 282 villages selected, and a total of 7,270 people were screened for palpable nodules. This report did not indicate the sex or age of people, only that is was conducted in “randomly chosen adults”. In the selected villages of Nampula, Manica and Sofala no onchocercomas were found. Only 50 positive cases were found in thirty villages; 2 in Inhambane and Tete, 11 in Niassa, 18 in Zambezia and 19 in Cabo Delgado. Nodule incidence was between 1.0–9.0 % in 28 villages and between 10.0–15.2 % in 2 villages. The REMO report concluded that the positive cases of *Onchocerca* nodules from Zambezia could be a spillover of infection from the neighbouring Malawi focus. It was concluded that the Niassa focus could be independent of the Tanzania side, but that this needed further investigation [31].

The second REMO [30] took place six years later (2007) to re-evaluate the status of the disease in relation to the findings of 2001 study that rated the disease as hypoendemic [31]. Based on the 2001 REMO survey that demonstrated the existence of the disease, and suitable ecology, combined with the continuous population migration mentioned previously this study was restricted only to Niassa, Cabo Delgado, Zambezia and Tete. A total of 114 villages were selected, amongst which 97 were successfully screened. A total of 3,780 consenting adults were examined for *O. volvulus* nodules. Most of the participants (86.96 %) were male. The results demonstrated that 61 people (1.6 %) from the four provinces showed characteristic onchocercomas. Of the 40 villages with people positive for *O. volvulus* nodules, a nodule rate of 1–9 % was observed and only two had a nodule rate varying between 10 and 19 %. Three villages (Mambucha, Vulalo and Matege) were visited in Niassa Province, and although Noormahomed found high nodule rates (34 %, 51 % and 38 %, respectively) in 1998, only one infected person (out of 30 examined) was found in Vulalo.

The REMO report [30] concluded that onchocerciasis exists in Mozambique but is hypoendemic and indicated that in Niassa there seems to be a focal area between Maniamba and Lussimbesse, where a small cluster of villages had positive cases. Based on these results this REMO study recommended further entomological studies and parasitological investigation, including standardised skin snips to determine the extent of the infection, and envisaged some clinic based treatment with ivermectin for the present cases and others that may be detected.

## Discussion

The presence of autochthonous and imported onchocerciasis cases was evidenced in five provinces of Mozambique: Niassa, Cabo Delgado, Zambezia, Tete and Inhambane. The four diagnostic studies demonstrated important differences in design which can potentially explain the divergence in findings. The use of skin biopsy and a symptom screening questionnaire combined with nodule rate incidence in the first two surveys (1997/98) may have captured even atypical or subacute clinical presentations. Both REMO studies relied solely on detection of palpable *O. volvulus* nodules. Taking into consideration the results of Noormahomed in 1998, at least 38.7 % of cases would have remained undiagnosed in REMO studies. Measurement error in nodule prevalence has been subsequently confirmed in larger studies by other authors detecting a significant variation between geographical regions [32]. Skin biopsy, although superior to palpation, is also insensitive, as has been shown using additional qPCR [23].

It is important to emphasise that between the two REMO (six years apart), the prevalence of nodules detected had more than doubled (from 0.69 % to 1.6 %). In both interventions, positive cases were in the villages of Niassa, Zambezia Cabo Delgado and Tete. The question that arises from these results, after a further eight years have almost elapsed is: “What is the current prevalence in these villages?”

The culture and local languages of northern Mozambique and Malawi are similar, no visa is needed and immigration control is lax. The social and cultural ties of the populations in the Northern provinces, and their kith and kin in the neighbouring countries, increase the likelihood that the disease will establish itself in Mozambique. A great number of refugees left Mozambique for Malawi during the civil war (1976–1992), and many have since returned. Some may have lived in endemic areas where they were exposed to and likely infected by *O. volvulus*. The return of refugees and the mixing of populations from both countries has meant that infected people, whether due to autochthonous or allochthonous exposure to onchocerciasis, are found in Mozambique.

After some years of controlling leprosy and declaring it no more a public health problem in 2010, Mozambique is now witnessing an increase in the number of cases, especially in the Nampula Province [33]. It is surprising that Nampula was reported in the first REMO as not having onchocerciasis, though this province has the most cases of leprosy in the country, with a prevalence of 0.006 % among inhabitants in 2014, (0.013 % in some districts), and no clear reason has emerged to explain this high prevalence (data from the Ministry of Health of Mozambique 2014). It is possible that some of these cases may in fact represent unrecognised cases of onchocerciasis that fail to improve despite being subjected to treatment against leprosy. Some health workers reported “atypical” cases of leprosy, in which patients present with skin sensitivity and no improvement after completing the leprosy treatment. We believe that these cases might be misdiagnosed cases of onchocerciasis. Those patients with a diagnosis of leprosy may also have been absent from the study population since stigmatisation from their leprosy diagnosis might lead to isolation. The provinces of Zambezia, Cabo Delgado, Niassa and Manica are also the most affected ones with leprosy. Those provinces, except Manica, are the ones where REMO 2 detected cases of onchocerciasis, with the possibility that some patients with nodules and skin disorders were confused and treated as leprosy patients. The two conditions may even present at the same time, in which case the diagnosis will be of lepromatous leprosy, because onchocerciasis is not recognised by health professionals.

Scabies is another condition that generates dermal changes such as an irresistible itch. Patients diagnosed with scabies, but who do not respond to treatment with hexochloride benzene, may actually be infected with onchocerciasis. The possible relationship between onchocerciasis with epilepsy also needs to be investigated in Mozambique.

During our first screening study, most patients were unaware of the cause of the disease while others strongly believed that it was a disease caused by spirits. This belief was reinforced as secondarily infected nodules resolved with traditional treatment. Visual impairment characteristic of onchocerciasis was not a prevalent finding, however in the study of 1998 (Table 1) 102 patients with positive skin biopsy reported visual impairment. A family of two with this symptomatology and microfiladermia was found during the first study although no member was blind.

Unresolved issues remain concerning transmission, especially near to the breeding sites of autochthonous *Simulium* spp. The results of a geostatistical analysis of all the REMO data [34] showed clearly that Mozambique has borders with two high-risk areas of onchocerciasis located in Tanzania and Malawi. The authors of this analysis indicated textually: “In Gabon and Mozambique only 0.3 % of the surface was classified as a high risk area. In both cases, it concerned a narrow border area where no REMO surveys were done within the country itself but where the presence of a large endemic focus on the other side of the border in the neighbouring country suggests that there may be also some hyperendemic villages on the banks of the border river within Gabon and Mozambique. Except for those narrow border areas, the REMO data for these two countries do not indicate the presence of any high risk areas”. Obviously in the case of Mozambique reference of the experts should refer the Ruvuma River which marks the border with Tanzania. There are large and small rivers in the northern provinces of Mozambique with many potential breeding sites for blackflies. There are reports referring to the River Muloza as a vector-breeding site. The presence of onchocerciasis and the vector were demonstrated years ago in the districts of Mwanza, Thyolo and Mulanje (Malawi) [28, 29]. Mulanje is just over 40 km from the Mozambique border. Given that *Simulium* spp. can easily travel long distances it is likely that the vector crosses the border into Mozambique or breeds along that same border inside Mozambique.

## Conclusions

The studies analysed here provided evidence of the existence of onchocerciasis in Mozambique, although it is hypoendemic. As the rural population moves frequently from one place to another, it is possible that the distribution of the disease has changed since the last REMO performed in 2007. Based on the preliminary data, onchocerciasis may be present at least in five provinces of Mozambique. Targeted epidemiological surveillance is needed to monitor that the disease is not spreading to other provinces, given that the vector can fly long distances.

To date little is known about the epidemiology and clinical aspects of the disease in Mozambique or its transmission patterns. The risk factors need to be identified and validated, and vulnerable populations and geographic areas identified as priorities for further research and targeting of surveillance activities. Seroepidemiological and parasitological investigations, using antigen detection cards and standardised skin snips, should be carried out in focal areas to determine the extent of the infection. These measures will contribute to the entry of potential onchocerciasis patients into the health care system for appropriate treatment and care. There is no awareness of the clinical aspects of the disease, differential diagnosis with lepromatous leprosy and dermatitis caused by *Scabies scabiei* needs to be addressed by training health professionals in the recognition of this disease. It has been suggested that the infection by *O. volvulus* can be associated with epilepsy, and if so, a study would be required to determine the proportion of epilepsy sufferers that are infected. There is an ongoing program in Mozambique (2012–2016) for controlling neglected tropical diseases, including schistosomiasis, lymphatic filariasis and soil transmitted helminths. Mass drug administration is conducted every year to more than 500,000 people [35]. We must prioritise studies designed to understand clinical and epidemiological aspects of onchocerciasis and its distribution, the efficacy of mass drug treatment, and if there is emerging ivermectin resistance. Entomological samples should be taken in Cabo Delgado, Niassa, Nampula, Zambezia, Tete and Manica, in order to explore the existence or otherwise of anthropophilic black flies and studies should be carried out to ascertain their capacity as vectors. As was indicated in 2014 (44) the first step in the implementation of REMO in each country involved the identification of possible areas that are unsuitable for onchocerciasis transmission, and where, therefore, no further surveys were required. So, the identification in Mozambique of suitable and unsuitable areas for *Simulium* spp. breeding will be a priority. Future investigation should therefore establish whether or not the vectors breed in the rivers of Mozambique.

### Ethical approval

All studies described in this paper have been approved by the Ministry of Health Ethics Committee and by the Ethics Committee of the Faculty of Medicine, Eduardo Mondlane University in Mozambique. Informed consent was obtained from each participant.

## Abbreviations

APOC: African programme for Onchocerciasis control
CDTI: community directed treatment with ivermectin
REMO: rapid epidemiological mapping of Onchocerciasis
WHO: world health organisation
